# Haplotype allelic classes for detecting ongoing positive selection

**DOI:** 10.1186/1471-2105-11-65

**Published:** 2010-01-28

**Authors:** Julie Hussin, Philippe Nadeau, Jean-François Lefebvre, Damian Labuda

**Affiliations:** 1 Bioinformatics Program, Department of Biochemistry, Université de Montréal, Montréal, Québec, Canada; 2 Research Center, Hôpital Sainte-Justine, Montréal, Québec, Canada; 3 Department of Pediatrics, Université de Montréal, Montréal, Québec, Canada H3T 1C8

## Abstract

**Background:**

Natural selection eliminates detrimental and favors advantageous phenotypes. This process leaves characteristic signatures in underlying genomic segments that can be recognized through deviations in allelic or haplotypic frequency spectra. To provide an identifiable signature of recent positive selection that can be detected by comparison with the background distribution, we introduced a new way of looking at genomic polymorphisms: haplotype allelic classes.

**Results:**

The model combines segregating sites and haplotypic information in order to reveal useful data characteristics. We developed a summary statistic, *Svd*, to compare the distribution of the haplotypes carrying the selected allele with the distribution of the remaining ones. Coalescence simulations are used to study the distributions under standard population models assuming neutrality, demographic scenarios and selection models. To test, in practice, haplotype allelic class performance and the derived statistic in capturing deviation from neutrality due to positive selection, we analyzed haplotypic variation in detail in the locus of lactase persistence in the three HapMap Phase II populations.

**Conclusions:**

We showed that the *Svd *statistic is less sensitive than other tests to confounding factors such as demography or recombination. Our approach succeeds in identifying candidate loci, such as the lactase-persistence locus, as targets of strong positive selection and provides a new tool complementary to other tests to study natural selection in genomic data.

## Background

The role of positive selection in the evolution and local adaptation of modern humans has been extensively studied using DNA variation data [1-6]. The increasing availability of such data led to the development of new statistical methods to detect signatures of natural selection along DNA sequences. As these techniques use and analyze DNA diversity in different ways, the overlap between the reported candidate loci under selection is relatively low [6]. Indeed, different summary statistics may capture different types of selection events. In addition, signatures may differ depending on the sequence context, time and strength of selection [4]. In the context of human evolution, it is particularly interesting to look for recent selection events resulting from local adaptations. These should have left signatures of incomplete selective sweeps in the human genome, where the selected allele dominates but is not yet fixed in a population. Loci affected by such selective events are likely to be of functional importance and responsible for inter-individual differences in genetic susceptibility to disease and/or to therapeutic outcome. Most of the early techniques to detect selection from DNA variation analyze allelic frequency spectra of individual polymorphic sites [7-10]. Newer methods look at haplotypes, their frequencies and length to capture those with extended linkage disequilibrium (LD), suggestive of a rapid and recent rise in population frequency and thus plausibly due to selection [1-3]. Other tests, such as that of Fu [11] or Depaulis and Veuille [12] propose to integrate information on haplotypes and their underlying sites. However, these tests are inadequate in the presence of recombination.

In order to combine information on alleles of single nucleotide polymorphisms (SNPs) with that of the resulting haplotypes, we propose to plot haplotype allelic classes (HACs) that group haplotypes of the same mutational distance from a predefined reference haplotype [13]. This distance, also called HAC, is calculated as the count of allelic differences between the reference and the individual haplotypes in the sample. The HAC distribution (i.e. the number of haplotypes belonging to each class) expected under neutrality can be evaluated by computer simulations. If one finds, in a sample, a significant deviation from the neutral HAC distribution, it may be concluded that the genetic variation observed in the sample is not neutral.

A critical point is the choice of the reference haplotype defining the classes. This haplotype does not have to exist in the sample and can be chosen to suit a particular application. If we aim to study patterns of genetic variation and haplotype diversity in a population sample, the ancestral haplotype would be an appropriate reference haplotype [13]. The HAC of a given haplotype would thus correspond to the number of non-ancestral (derived) alleles it carries, ranging from zero to the total number of SNPs within the analyzed DNA sequences. Under an incomplete selective sweep model, haplotypes carrying a positively selected allele on its way to fixation are very likely to also carry a large proportion of major frequency alleles of the accompanying SNPs [5]. It is, therefore, practical to define as a reference a haplotype carrying only major frequency alleles of its constituting SNPs. This major-allele-reference haplotype (MARH) is expected to be structurally close to haplotypes carrying a positively selected allele. Using the MARH, the HAC of a given haplotype corresponds to the number of minor alleles it carries. A selective sweep is expected to favor haplotypes similar to the MARH and narrow HAC distribution with respect to neutral distribution. Therefore, we propose that HAC-derived statistics should be helpful in identifying selection events using genetic diversity data.

In this paper, we present *Svd*, the first summary statistic based on HAC distribution intended to detect ongoing selective sweeps. The resulting test can be used on a specific DNA region or to scan larger sequences using a sliding window approach. It appears less sensitive than other tests to confounding factors such as changes in population size or recombination. We successfully tested our approach using the lactase persistence locus on human chromosome 2, known to be under recent positive selection in a range of human populations [14-17].

## Methods

### Statistical Framework

#### Model

To evaluate the likelihood that a given SNP is affected by an ongoing selective sweep, we considered separately each of its two alleles. This SNP is referred to as the evaluated segregating site. We compared the HAC distribution of all haplotypes carrying the major allele of the evaluated site to the distribution of the remaining haplotypes carrying the minor allele. In order to compare these distributions, we considered their variances. For a neutrally evolving sequence, the spread of both distributions is expected to be a function of the frequency of the evaluated allele, the extent of the associated haplotypes and the recombination rate. When a sequence evolves under a positive selection, the selected allele rises in frequency. It drags behind all alleles of adjacent SNPs that are carried on the same haplotype, a process known as genetic hitchhiking [18]. Hence, the HAC distribution of haplotypes carrying the selected allele (or a linked hitch-hiked allele) will be tight and characterized by low variance. At the same time, the other allele would be expected to occur on a number of haplotypes with a broader HAC distribution, i.e., greater variance.

#### Variance Estimator

Since a probability distribution for HACs has not been theoretically derived, the variance *V*(HAC) has to be estimated. Let *n *be the number of sequences in the sample, *h*_*i *_(for *i *= 1..*n*) be the HAC of sequence *i*, and  be the empirical mean of the *h*_*i*_, then

is a consistent and asymptotically normal sample estimator for *V*(HAC).

#### *Svd *- a Statistic based on the HAC Variance Difference

We present a summary statistic developed to be computed independently at each SNP. For any evaluated SNP *k*, the *n *sampled sequences are divided into two sub-samples: the sub-sample *R*_*k*_, containing the haplotypes carrying the major allele (present on the MARH at SNP *k*) and the sub-sample *r*_*k*_, containing the remaining haplotypes. We can then compute

where  and  are the variance estimators for the sub-samples *R*_*k *_and *r*_*k*_, respectively. Under neutrality, *vd*_*k *_is expected to be close to zero, when *R*_*k *_and *r*_*k *_contain a similar number of sequences, or negative, when *R*_*k *_contains significantly more sequences than r_k_.

When the selected allele reaches major frequency due to positive selection, the speed of this frequency rise leaves little time for the carrier haplotype to diversify by mutation or recombination. The HAC distribution for *R*_*k *_is then expected to be tight and close to 0, making  particularly small. Hence, *vd*_*k *_is expected to be positive when computed for a selected SNP and/or its linked sites.

The *vd*_*k *_values should be normalized, in order to be independent of haplotype length, to the number of the contributing SNPs *S*. We can demonstrate (see Additional File 1) that the HAC variance is in O(S). We thus obtain a normalized difference of variance estimators by dividing *vd*_*k *_by *S*. Furthermore, because we only consider cases when selection drives new alleles to major frequencies, whereas high frequency ancestral alleles are of little interest, the normalized *vd*_*k *_values are weighted by the derived allele frequency of SNP *k*, *f*_*d*, *k*_, to obtain the following summary statistic:

#### Statistical Test of Neutrality using *Svd*

*Svd *can be used as a decision variable for a test that could statistically distinguish a site evolving under neutrality from one subjected to ongoing positive selection. Neutrality is rejected when *Svd *is superior to a critical value. For all subsequent analyses, the critical value *c *of the test is defined as Pr(*Svd *>*c*|neutrality) = *p*, with *p *= 0.05. The detection power represents the sensitivity of the test, i.e., the probability of having *Svd *>*c *when a selective sweep is in progress.

### Test Validation Using Simulations

Simulated data under various scenarios is used to compute the distribution of *Svd *and evaluate its detection power to find signatures of ongoing positive selection. We simulated DNA sequences under a wide range of neutral and selection models. Each simulated data set contained 1,000 sample replicates of *n *= 50 sequences obtained with a population mutation rate Θ = 223, which on average leads to ~1,000 SNPs per sample of 50 sequences, under selective neutrality with constant population size [19]. The simulated datasets were evaluated using *Svd *and three other statistics: the unstandardized version of LD-based statistic *iHS *[2] and two site-frequency-spectrum statistics, Tajima's *D *[8] and the normalized version of Fay and Wu's *H *[7,10]. All statistics were calculated for haplotypes of fixed length *S *= 51, with the evaluated site located at their central position. For *Svd*, additional lengths were examined (S = 26, 51, 201, see Table 1).

**Table 1:** *Svd *power to detect selection in the context of various population scenarios.

Population model parameters	**Window size **(*S*)	Detection Power
Default	25	0.74
	50	0.81
	200	0.84

× population size	50	0.8
2 x population size		0.91

Constant recombination rate	50	0.68
Weak recombination hotspot		0.67
Strong recombination hotspot		0.65

Coalescence simulations under selective neutrality were carried out using the ms program [20]. In a standard scenario, population evolves for 4,000 generations without recombination. In a population bottleneck scenario, the same population evolves for 3,660 generations, experiences a 95% reduction in size during 80 generations and recovers for subsequent 260 generations (see Additional File 1). At demographic expansion, a population of *N*_*e *_= 500 grows to *N*_*e *_= 1,000 in the last 300 generations (see Additional File 1). Recombination was tested under the standard scenario with a population recombination rate *ρ *= Θ/2, kept constant along the sequence.

SelSim [21] was used to simulate sets of replicates under an ongoing selective sweep. In a default selection scenario, a population evolves under the standard scenario with the evaluated SNP brought to a frequency of *f *= 0.75 by the ongoing positive selection with a selection coefficient of *s *= 0.15. Small and large population selection scenarios were tested, where a population of *N*_*e *_= 500 and *N*_*e *_= 2000, respectively, evolved under the default selection scenario. Recombination was tested under the default selection scenario with a population recombination rate *ρ *= Θ/2 kept constant along the sequence and in the presence of hotspots. In the latter case, the background rate is again *ρ*_*b *_= Θ/2 with hotspots rate *ρ*_*HS *_corresponding to 10 *ρ*_*b *_(weak hotspot) and 100 *ρ*_*b *_(strong hotspot). Hotspots are located 2 Kb downstream of the evaluated site. In addition, samples for a range of values of *f *= 0.6, 0.7, 0.75, 0.8, 0.9 and *s *= 0.05, 0.15, 0.5 were also simulated.

### Ascertainment Bias and Haplotype Phasing

In some ascertainment protocols, SNPs are reported only if they have some minimum frequency in the sample. Since sites with a minor allele frequency (MAF) below 0.05 are considered more likely to reflect sequencing errors and less useful in genome-wide mapping, they were typically excluded from genotyping chips. To approximate such situations, singletons and doubletons were removed from the simulated replicates (with *n *= 50, these SNPs have a MAF below 0.05). In addition, we recreated an ascertainment scheme involving the identification of SNPs in a smaller sequencing panel consisting of *m *chromosomes and genotyping them in a larger panel of size *n*. To evaluate the impact of the sequencing panel size, different values of *m *were considered: *m *= 4, 8, 12, 16, 20, 26, 32, 38, 44 and 50 (at *m *= 50, there is no ascertainment bias). The ascertainment procedures are applied to each replicate simulated under the default selection scenario. To recreate the effect of haplotype phasing, for each replicate of a simulated dataset, we randomly assigned *n *= 50 sequences to 25 individuals. We then resolved the resulting genotypes back to haplotypes using the fastphase program [22]. The *Svd *statistic was then computed on haplotypes of length *S *= 50, 200, 400, 600 and 800, centered on the evaluated site. This procedure was applied to the set of replicates simulated under the default selection scenario.

### Detection Power

To assess the detection power of *Svd*, *iHS*, *D *and *H *under different selection scenarios, we needed to determine critical values at *p *= 0.05 for each set of parameters. These critical values were obtained by computing the statistics on datasets simulated under the same scenarios, with identical ascertainment and haplotype reconstruction procedures and with identical parameters except for the selection coefficient, which was set to *s *= 0. The critical *Svd *value *c *was determined for each scenario so that the proportion of *Svd *values greater than or equal to *c*, at *s *= 0, was exactly 0.05.

### Application to Data

#### Genotypes

Experimental data were from the HapMap project, Phase II Release 21a [23]. The Japanese (JPT) and Chinese (CHB) samples were considered together as the East-Asian (ASI) sample of 89 unrelated individuals. The West European (CEU) sample and the Yoruba from Nigeria (YRI) sample contain 60 unrelated individuals each. The phased haplotype data were taken directly from the BioMart HapMap browser http://hapmart.hapmap.org/BioMart/martview, which no longer gives access to the Phase II Release 21a dataset. This dataset is currently available from the HapMap ftp site ftp://ftp.ncbi.nlm.nih.gov/hapmap/. The chimp allele, or the macaque allele when the chimp allele was unavailable, was used as a proxy of the ancestral allele of a human SNP, found through the UCSC table browser http://genome.ucsc.edu/cgi-bin/hgTables?command=start. When both the chimp and macaque orthologous alleles were unavailable in the UCSC database, such SNPs were discarded.

#### Scan and Candidate Approach

We used a sliding window approach with different window lengths to analyze the entire chromosome 2 in ASI, CEU and YRI. The number of SNPs analyzed was 221,956, 206,665 and 252,249, respectively. The window of fixed length *S *slides one SNP at a time. We assigned *p*-values to each SNP according to the empirical distribution of *Svd *values, computed for all SNPs of chromosome 2.

In addition, we analyzed the lactase persistence locus in CEU, where we considered 26 polymorphic sites (rs IDs are listed in Additional File 2, Table S1) from the MCM6 gene in the genomic region Chr2:136424478..136459810. To measure confidence in inference of selection in this genomic region, for each SNP we evaluated its associated *p*-value based on a simulated distribution of *Svd *values (see below).

#### Replicates Matching the MCM6 Locus

To assign *p*-values to the observed CEU data, we simulated a set of 1000 replicates, with 120 chromosomes, at the population mutation rate Θ = 223. The evaluated SNP in all replicates was under positive selection at *s *= 0.15, assuming current *f *= 0.78, which corresponds to the frequency of the MCM6 T variant (rs4988235) in CEU. To model SNP ascertainment, we used a rejection sampling, as described by Voight and collaborators [2], to modify the simulated frequency spectrum to correspond to the observed frequency spectrum of SNPs in chromosome 2. To match the MCM6 locus in CEU, haplotypes of 26 SNPs were chosen in such a way that the 8^th ^SNP of each replicate is the one under positive selection. *P*-values were estimated by comparing the *Svd *values computed from experimental data to the *Svd *distribution obtained by simulation.

## Results

### Distribution of *Svd *Values

**Figure 1:**
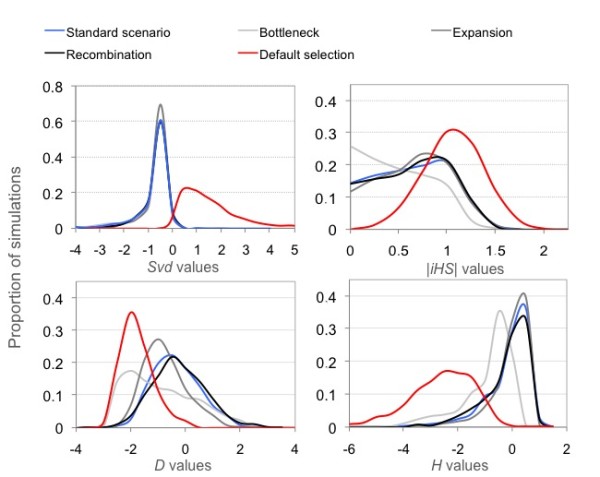
**Simulated distribution of *Svd, iHS*, *D *and *H *in neutrally evolving populations and under an ongoing selective sweep**.

The distribution of *Svd *values obtained under the default selection scenario is sharply different from distributions observed under a range of neutral scenarios, which are almost identical to each other (Figure 1A). This difference is less dramatic in the case of *iHS*, *D *and *H*, indicating relatively poorer discrimination of selection by these three statistics. Figure 1 shows only a small overlap between *Svd *values computed under selection and under other scenarios, supporting further the relative robustness of *Svd*.

### *Svd *Power to Detect Ongoing Positive Selection

Under the default selection scenario, the detection power of *Svd *at *p *= 0.05 is 0.81 (Table 1). Its detection power at different false discovery rates (FDR) outperforms the three compared statistics at FDR > 0.05 (Additional File 2, Figure S1). On the other hand, *Svd *is less efficient than *iHS *at FDR < 0.05 and its performance becomes comparable to *D *at even lower FDRs. Overall, *iHS *appears to have the highest specificity, whereas *Svd *has the highest sensitivity with the detection power reaching 0.95 at FDR = 0.1.

**Figure 2:**
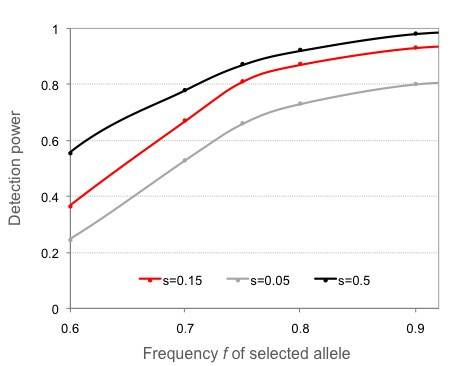
**Power of *Svd *to detect ongoing selective sweeps**.

**Figure 3:**
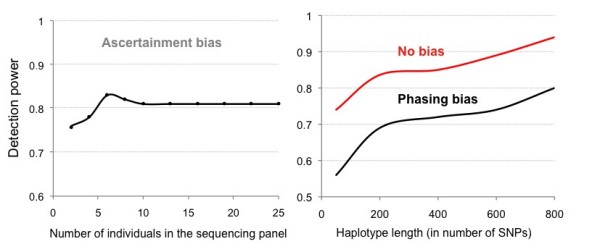
**Impact of experimental bias on the detection power of *Svd***.

The detection power of *Svd *increases with haplotype length (i.e. window size) and when the population size is greater (Table 1). It decreases when the analyzed segment undergoes recombination and in presence of recombination hotspots. The power of the test increases with the increasing strength of selection *s *and/or the increasing frequency of the selected allele *f *(Figure 2). We observed a small effect of the ascertainment bias introduced by genotyping SNPs, which were found in the sequencing panel consisting of a smaller number of individuals than the genotyped sample (Figure 3A). Ascertaining SNPs in less than 5 out of 25 genotyped individuals decreased the detection power. It remained practically constant when half or more of the genotyped individuals were used in the ascertainment. A slight decrease in the detection power following 6 individuals (Figure 3A) can be explained by an increased number of rare SNPs that are eventually genotyped due to an increasing number of individuals in the sequencing panel. As a result, the compared HAC distributions became noisier. Greater proportion of practically non-informative SNPs in the analyzed haplotypes effectively lowers the window size and thus affects the detection power. Consistently, the removal of the singleton and doubleton SNPs from the analyzed replicates increases the detection power from 0.81 to 0.88. The determination of haplotype phase experimentally is prohibitively expensive, whereas it is done relatively efficiently using statistical methods such as the PHASE and fastphase algorithms [22,24]. However, as these algorithms tend to cluster the sampled sequences together into groups of similar haplotypes, the phasing procedure is expected to narrow the HAC distribution. This may reduce *Svd *values and decrease the detection power of the test. An important drop in detection power, from 0.81 to 0.56, was observed following phasing by fastphase (Figure 3B). Because of the nature of our test, where only mutational distance from the MARH matters, using longer haplotypes can compensate the decrease in power due to phasing (Figure 3B).

### Application to the Data

**Figure 4:**
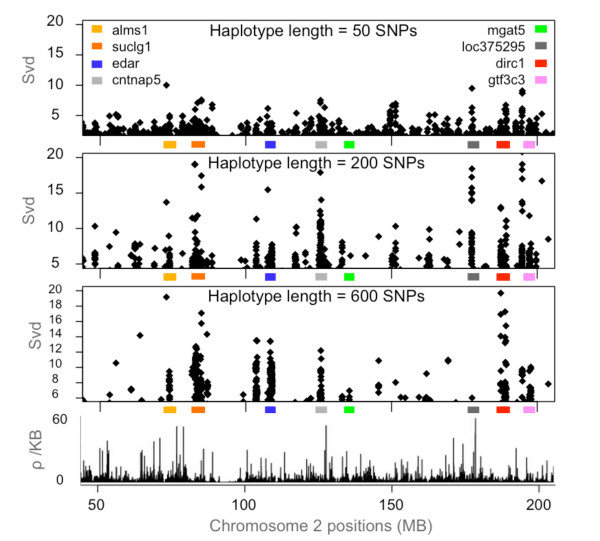
**Top *Svd *values in the 50- to 200-Mb region of the human chromosome 2, based on the sliding window scan in the ASI population sample, using different haplotype length (window size)**. For clarity, only the values in the 99^th ^percentile of the computed *Svd *> 0 are plotted. Bottom plot shows variation in the fine-scale recombination rate *ρ *estimated using InfRec [35]. The colored boxes indicate the location of six loci previously identified as targets of selection [25-27]. The gray shading boxes indicate the location of two new loci, where clustered signals found by our approach suggest positive selection as well.

Using HapMap data, we analyzed *Svd *distribution along chromosome 2. Figure 4 presents results of this analysis for a 50- to 200-Mb segment of chromosome 2 in the ASI sample. For clarity, it only shows the top 1% of the positive *Svd *values. When these top SNPs occur in clusters, it suggests that an ongoing selective sweep can be taking place in the underlying genomic regions. Interestingly, the clusters we observed include six loci (ALMS1, SUCLG1, EDAR, MGAT5, DIRC1 and GTF3C3) that were earlier proposed as positively selected by others [25-27]. In most instances, both the intensity and resolution of these clusters increase in relation to the window size from *S *= 50 to *S *= 200 and even above. We note, however, that some signals fade with the increasing window size in the range of the examined window sizes. This is the case of the LOC375295 adjacent cluster located at 177 Mb. This behavior is likely a function of the extent of LD surrounding the selected site, reflecting either the age of a selective sweep or the local intensity of recombination, or both (Figure 4 and Additional File 2, Figure S2). Another clustered signal, seen at all window sizes, suggests positive selection in the 124- to 125-Mb region. Interestingly, this region contains the CNTNAP5 gene of the neurexin family involved in cell contacts and communication in the nervous system. Table 2 summarizes loci previously identified by other studies that also display strong signals of ongoing positive selection in the *Svd *scan of chromosome 2. We also reported the *p*-values of the iHS statistic, which successfully identified 4 of the 10 loci reported.

**Table 2:** Results of *Svd *scan in ten chromosome 2 genes under positive selection according to previous reports

		CEU			ASI			YRI		
Genes (chr2)	Ref studies	Most significant SNP	*p*value(*S*)	iHS *p*value	Most significant SNP	*p*value(*S*)	iHS *p*value	Most significant SNP	*p*value(*S*)	iHS *p*value
ALMS1	[25]	-	-	-	rs11126402	5.13·10^-4^ (400)	0.755	-	-	-

EDAR	[25-27]	-	-	-	rs17036146	1.45·10^-5^ (800)	**1.05·10^-3^**	-	-	-

DIRC1	[26]	-	-	-	rs7578063	9.68·10^-6^ (400)	0.608	-	-	-

GTF3C3	[25]	rs10163352	1.85·10^-3^ (400)	**6.15·10^-3^**	rs12989157	1.88·10^-4^ (200)	**9.74·10^-3^**	-	-	-

MCM6	[2]	rs4988235	7.88·10^-4^ (800)	**3.61·10^-4^**	-	-	-	-	-	-

LRP1B*	[26]	-	-	-	-	-	-	rs10194564	1.67·10^-4^ (600)	0.209

MGAT5	[25]	rs1561277	3.15·10^-5^ (200)	6.72·10^-2^	rs7608637	1.46·10^-3^ (200)	0.753	-	-	-

SLC3A1	[27]	-	-	-	-	-	-	rs1067321	5.55·10^-5^ (600)	0.149

ADCY3	[2]	-	-	-	-	-	-	rs713587	1.11·10^-4^ (800)	**5.98·10^-3^**

SUCLG1*	[25]	rs10210248	3.60·10^-5^ (400)	0.787	rs6721249	6.44·10^-4^ (800)	3.82·10^-2^	-	-	-

Table reports rs ID of SNPs showing the most significant signal as indicated by its *p*-value at window size indicated in parenthesis (* the signal appears in the upstream region of the gene). For each significant signal, the *p*-value computed for iHS in the gene and population by the Haplotter web tool is reported (significant values at p < 0.01 are written in **bold**).

**Figure 5:**
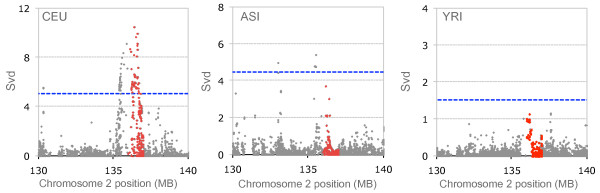
**Positive *Svd *values in a 10-Mb region of chromosome 2**. Plots of *Svd *> 0 for the three HapMap populations using a window size of 800 SNPs. *Svd *values plotted above the dashed blue lines are in the 99^th ^percentile of all positive values computed for the whole chromosome 2 in each of the population samples. The 1-Mb segment containing the LCT and MCM6 genes is plotted in red. A strong and clear signal of positive selection is found in this region in CEU, while no signal is detected in the two other populations.

**Figure 6:**
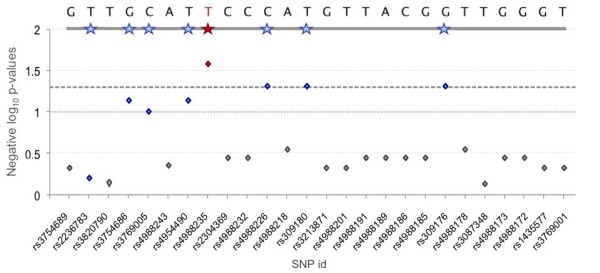
**Using the *Svd *test at the MCM6 locus**. Plot of the negative log *p*-values for the 26 SNPs in the MCM6 locus in CEU. The reference major allele frequency haplotype is shown on top. Stars indicate derived alleles and the red star corresponds to the T-13910 mutation (rs4988235), believed to be responsible for the trait in Europeans [28]. *P*-values were obtained as described in Methods and the dotted and dashed line delimit *p *= 0.1 and *p *= 0.05 cutoff, respectively.

Comparing signals between populations can help validate targets of selection. Figure 5 compares positive *Svd *plots for the three HapMap population samples in the 130- to 140-Mb region of chromosome 2. Its smaller segment which contains two neighbouring genes, lactase (LCT) and MCM6, is highlighted in red. As shown, no single SNP reaches the top 1% of positive *Svd *values in ASI and YRI. In contrast, a strong *Svd *signal, consistent with the ongoing positive selection is observed in the CEU population. A transition from C to T (rs 4988235) located in the MCM6 gene 13910 bp upstream of the LCT initiation codon, is known to be responsible for the lactase persistence phenotype in Europeans [28]. Our results above as well as those obtained using LD-based methods all indicate the effect of ongoing positive selection in this region [1,17]. We computed *Svd *for each of the 26 SNPs found in the MCM6 locus as shown in Figure 6. *Svd *values were computed for each evaluated site in the context of the haplotype consisting of the 25 remaining SNPs. The reported *p*-values were obtained from simulations separately for each of the observed *Svd *values (see Methods). The 13910T lactase persistence variant is found on a haplotype carrying 18 ancestral and 8 derived alleles and this particular haplotype turns out to be the reference haplotype, because all its alleles are major. A *p*-value of 0.026 obtained for the C → T-13910 polymorphism is consistent with the role of its T allele in lactase persistence in Europeans.

## Discussion

The neutral theory of molecular evolution [29] recognizes genetic drift as the main force shaping genetic variation. However, many recent studies suggest that substantial portions of the human genome have evolved under positive selection [30]. Selected loci can cause changes in the frequency of genetically linked sites remarkably similar to fluctuations caused by genetic drift, as Gillespie's model of genetic draft suggests [31]. This means that if there are many genes undergoing partial selective sweeps in the human genome, genetic variation might be shaped by selective forces acting on adaptive mutations and not mainly by genetic drift. To test whether genetic variation should be interpreted in the light of models of draft rather than drift, it seemed that a good strategy would be to develop a statistical test specific for detection of incomplete selective sweeps.

In this paper, we have presented a novel intuitive and computationally efficient statistical test based on *Svd*, a statistic specifically created to look for genomic signatures of strong incomplete selective sweeps. When developing this statistic, we found it useful to start by displaying genomic diversity data in histograms of haplotype allelic classes that capture information on haplotype diversity combined with that on the contributing SNPs. In this way, HACs provide an interesting framework to developing summary statistics as convenient substrates to develop new neutrality tests.

The *Svd *statistic is based on the allelic variability of SNPs and the resulting haplotypes and on the expected different apportionment of these between the selected allele and its complementary allele for the site under sweep. It is thus likely that it behaves differently when compared with other statistics such as *iHS*, *D *or *H *and tends to be less sensitive to demographic changes. While our simulation experiments were based on a restricted set of parameters, they illustrate the fact that the *Svd *test has good detection power and should perform well on a variety of population models. We demonstrated the potential of the *Svd *test, applicable to genomic data when using a sliding window approach, as shown by our analysis of the human chromosome 2 (Figures 4 and 5). To evaluate the statistical significance of the outcome of the test, we first used an empirical approach. We assigned *p*-values to concrete *Svd *values based on the empirical distribution of all *Svd *values obtained by scanning the whole chromosome 2 in the analyzed population sample. Subsequently, to validate a candidate locus, such as MCM6, we evaluated *p*-values of each of its SNPs by simulations taking into account any prior information we may have had on the locus itself and on the population in which the signal was found (recombination rates, allelic frequencies, demography, SNP-ascertainment protocol). A strong signal of ongoing positive selection in the lactase persistence locus is found only in the European-derived population. This result was expected. In Europe, cattle were domesticated 10,000 years ago and cultural habits associated with milk consumption may have been advantageous for individuals (nutritional benefit, improved calcium absorption [14]). Although the SNP with the strongest *Svd *signal, based on the *p*-value obtained by simulation, was already identified as associated with lactase persistence in European populations, our analysis demonstrates the great potential of the proposed method in detecting new candidate polymorphisms for association studies.

The majority of available genotyping datasets are biased in the choice of the genetic markers typed, because they were collected for use in linkage and association studies and the analysis of this data should focus on tests of overall diversity [4]. *Svd *can thus be applied to such datasets because computing HAC distribution provides a summary of overall haplotype diversity. In addition, the removal of rare SNPs from simulated data increases detection power, which suggests that the *Svd *test may perform even better on data with common SNPs than on data with rare and common variants. This can be explained by greater informativeness of common SNPs. Removal of rare SNPs increases the effective window size, thus increasing the detection power (Table 1, Figure 3A). In the case where a site under selection is not among SNPs that are genotyped, selection would still be detected by an *Svd *test through the surrounding linked SNPs, although the detection power may be decreased (data not shown).

Inaccuracy in haplotype inference is known to hamper the detection of signature of positive selection in genetic data and strategies to accurately infer haplotypes (e.g. using trio data) must be applied prior to using selection detection methods [32]. We observed, with simulation data, a loss of power of *Svd *selection test due to haplotype phasing, but the test remains conservative in the sense that phasing errors won't create false positive results. Using longer, and thus potentially more informative haplotypes can compensate this effect. Therefore, the use of large windows, in the range of hundreds of SNPs, could be recommended to increase the signal. If this works, it suggests that the selective sweep is relatively young or that its signature persists longer because of a relatively low local recombination rate. In other words, longer haplotypes appear to be more robust, but at the same time, are more sensitive to recombination and to the age of a genetic sweep. This explains why certain significant *Svd *signals may fade with the increasing window size. Different haplotype lengths are thus to be explored to scan the genome or a specific region of interest. Given the data and the recombination rates, we used a pre-treatment method to determine the "pseudo-optimal" haplotype length around each SNP to consider as a starting point and guide the practical analysis (see Additional File 1).

The idea behind the Svd statistic is very similar to the approach used to compute the *iHS *statistic [2]. The advantageous alleles favored by positive selection are generally found within large shared haplotypes where the level of diversity is reduced. These haplotypes contrast with the more variable haplotypes, which do not carry alleles under selection. With *iHS*, one can look at the decay of identity of haplotypes that carry a specific allele. With *Svd*, rather than looking at haplotype homozygosity, we contrast haplotypes carrying one or the other allele of the evaluated site. For haplotypes of 50 SNPs, at FDR = 0.05, *iHS *and *Svd *have the same detection power when the selected allele frequency is over 0.5 (Figure 1). When the selected allele frequency is under 0.5, *Svd *is not expected to find the signal whereas *iHS *can detect low frequency sweeps.

Furthermore, *iHS *outperforms Svd when FDR < 0.05. On the other hand, *Svd *power increases with haplotype length. Even if the edges of the selected haplotype are broken by recombination, the portion of originally selected haplotype still remain within the analyzed pool, portioned among different sequences. Using simulated data where the selected site is surrounded by one or two hotspots of recombination, we showed that *Svd *had a better detection power to identify signals of selection (Additional File 2, Table S2), because long range haplotype tests require intact haplotypes to remain in the population. Yet, recombination hotspots are expected every 50 Kb [33]. *Svd *can therefore be considered as a useful complement to long-range haplotype statistics in detecting signatures of recent positive selection.

## Conclusions

Different steps in the analysis of selection signatures proposed in this study can be modified, depending on the data and specific questions. Here, our reference haplotype was composed of predominant alleles in the population, but other reference haplotypes can be considered [13]. Other applications are also possible, such as the use of *Svd *to compare groups of haplotypes in case-control studies. Furthermore, because the HAC distribution is also sensitive to a complete selective sweep, an approach similar to the one proposed by Kimura and collaborators [34] to identify fixed loci under positive selection could be developed using HAC distribution instead of haplotype homozygosity.

## Authors' contributions

JH and JFL designed the statistical test. JH carried out the data analysis. JH and PN performed simulations. DL designed and coordinated the study. JH drafted the manuscript and DL and JFL revised it extensively. All authors read and approved the final manuscript.

## Supplementary Material

Additional file 1 **Supplementary details**. *Svd *normalization, simulation parameter choices, procedure to determine the haplotype lengths and method availability. Click here for file

Additional file 2 **Supplementary Figures and Tables**. Figures S1, S2 and Tables S1, S2. Click here for file
