# Supplementary material for: Haplotype allelic classes for detecting ongoing positive selection
Source: BMC Bioinformatics. 2010 Jan 28;11:65. doi: 10.1186/1471-2105-11-65 (PMC2831848; doi:10.1186/1471-2105-11-65)
Supplement: Additional file 1 — Supplementary details. Svd normalization, simulation parameter choices, procedure to determine the haplotype lengths and method availability. [file 1471-2105-11-65-S1.PDF]

Additional File 1 – Supplementary details

Svd normalization

For a sequence Q, let X_k (for $k = 1..S$) be the random variable which takes the value of 1 when the allele at site k in Q is different from the allele at site k in MARH, and 0 when these allelic states are identical. $X_k = 1$ with probability f_k (the frequency of the minor allele at site k), and $X_k = 0$ with probability $1 - f_k$.

HAC for sequence Q can be defined as the sum of the S Bernoulli random variables X_k, which variance is in O(S).

Simulation parameter choices

Due to the limitation of computational load in simulation programs (ms and selsim), we choose to set the default population size to $N_e = 1000$. Therefore, in our simulations, the default scaled selection coefficient $\sigma = 2N_e s = 300$ with $s = 0.15$, as in (Kimura, Fujimoto et al. 2007).

The 95% reduction bottleneck models a bottleneck starting when agriculture first appeared and lasting for about 2400 years ($\simeq$ 80 generations), followed by 7800 years of recuperation. The population growth models a population that doubles in the last 300 generations, corresponding to about 9000 years.

Procedure to determine the haplotype lengths

To determine the length of haplotype, we propose a pre-treatement method, to determine the pseudo-optimal window size around each SNP and guide the analysis in practice.

Using a sliding window approach, each SNP is placed at the center of a window whose size varies according to the local recombination rate. Let R be the maximum recombination rate found in the region to be scanned.

Let $S_{\min}$ and $S_{\max}$ be the minimum and maximum window sizes and $\sigma = \frac{S_{\min}}{S_{\max}}$.

Let r_i be the maximum recombination rate found in a window of size $S_{\max}$ centered on SNP i. The new window size $N(i)$ for SNP i is :

$$N(i) = S_{\max} * \left(\frac{(\sigma - 1) r_i}{R} + 1 \right)$$

When r_i is close to R, the window size is strongly reduced whereas it stays close to $S_{\max}$ when r_i is close to 0. This function allows us to take into account the variation in

recombination rates and, particularly, the presence of hotspots. However, in some cases, a recombination hotspot considered to reduce the window size will be located outside of the new reduced window. The haplotype considered in these cases is artificially reduced.

To prevent the procedure to arbitrarily reduce window sizes, we use the following algorithm :

1. Compute $N(i)$

2. Let d be the distance between SNP i and the site displaying the maximum recombination rate r_i. If r_i has been found outside of the new window of size $N(i)$, compute $N'(i)$:

$$N'(i) = S'_{\max} * \left(\frac{(\sigma' - 1) r_i}{R} + 1 \right)$$

where $S'_{\max} = 2(d - 1)$ and $\sigma' = \frac{S_{\min}}{S'_{\max}}$

3. $N(i)_{Final} = \max\{N(i), N'(i)\}$.

Here, a simple linear function is proposed but other functions can be used.

We scan the chromosome 2 with this method in the ASI population. For SNPs rs10166142 and rs6721249, whose Svd signals are presented in details in Fig. S2, there are no recombination hotspots in the near region, according to Infrec method (Lefebvre et al. 2008. Genetics). The pseudo-optimal window size given by our method with $S_{\max}$ = 800 and $S_{\min}$ = 50 are S = 780 for rs10166142 and S = 770 for rs6721249, which is close to $S_{\max}$.

SNPs rs1080392 (see Fig. S2) has a recombination hotspot in the near region. The pseudo-optimal window size given by our method with $S_{\max}$ = 800 and $S_{\min}$ = 50 is S = 540, which is smaller than $S_{\max}$.

Method availability

The methods used to compute window sizes and Svd values is implemented in programs written in JAVA, available at : http://www.iro.umontreal.ca/~hussinju/Svdtools.html
