# Supplementary material for: Haplotype allelic classes for detecting ongoing positive selection
Source: BMC Bioinformatics. 2010 Jan 28;11:65. doi: 10.1186/1471-2105-11-65 (PMC2831848; doi:10.1186/1471-2105-11-65)
Supplement: Additional file 2 — Supplementary Figures and Tables. Figures S1, S2 and Tables S1, S2. [file 1471-2105-11-65-S2.PDF]

Additional File 2 – Supplementary Figures and Tables

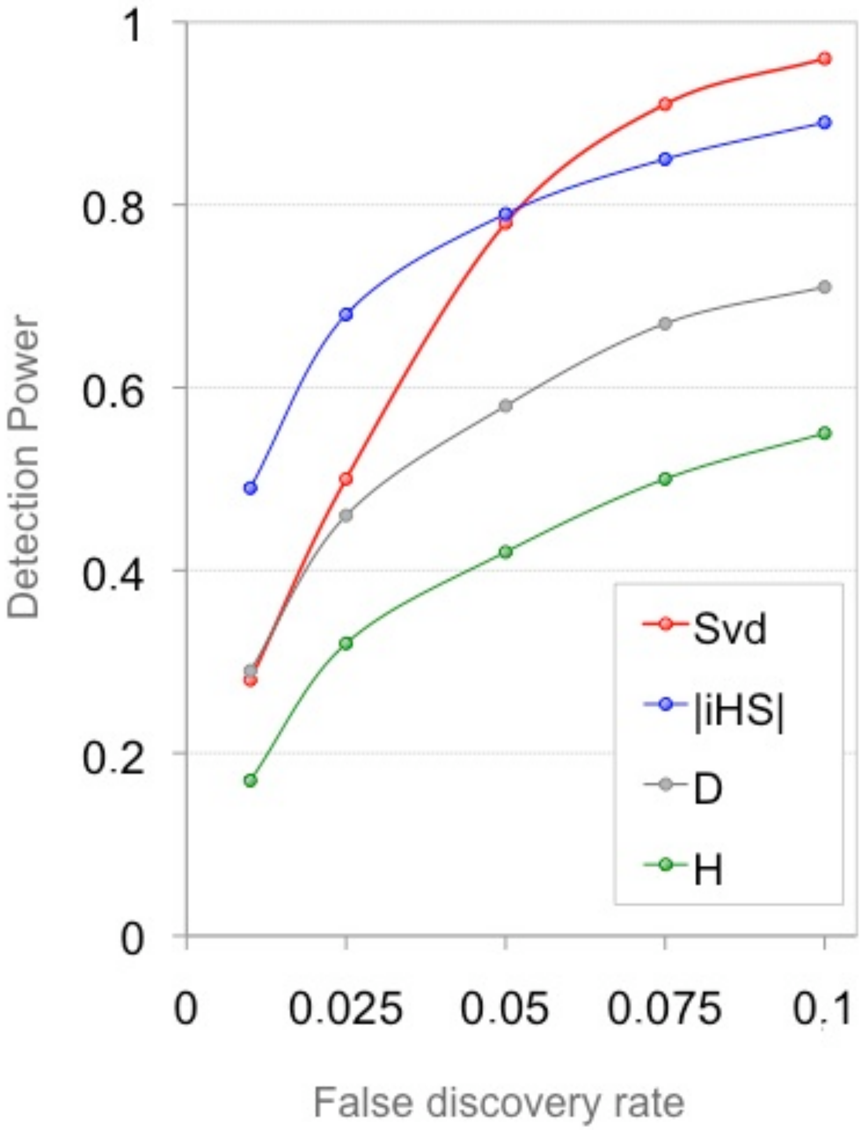

Figure S1 - *Svd* detection power compared to that of iHS, D and H statistics used to detect selection

Power to detect an ongoing sweep for the four statistics on simulated data at different false discovery rates. The statistics are computed on haplotypes simulated under the default selection scenario, on a centered evaluated SNP under selection in the context of 50 surrounding SNPs (S=51). Critical values are obtained using identical simulations with $s = 0$. The other statistics are: |iHS|, the absolute value of the unstandardized iHS statistic; D, the normalized value of Tajima's D; H, the normalized value of Fay and Wu's H.

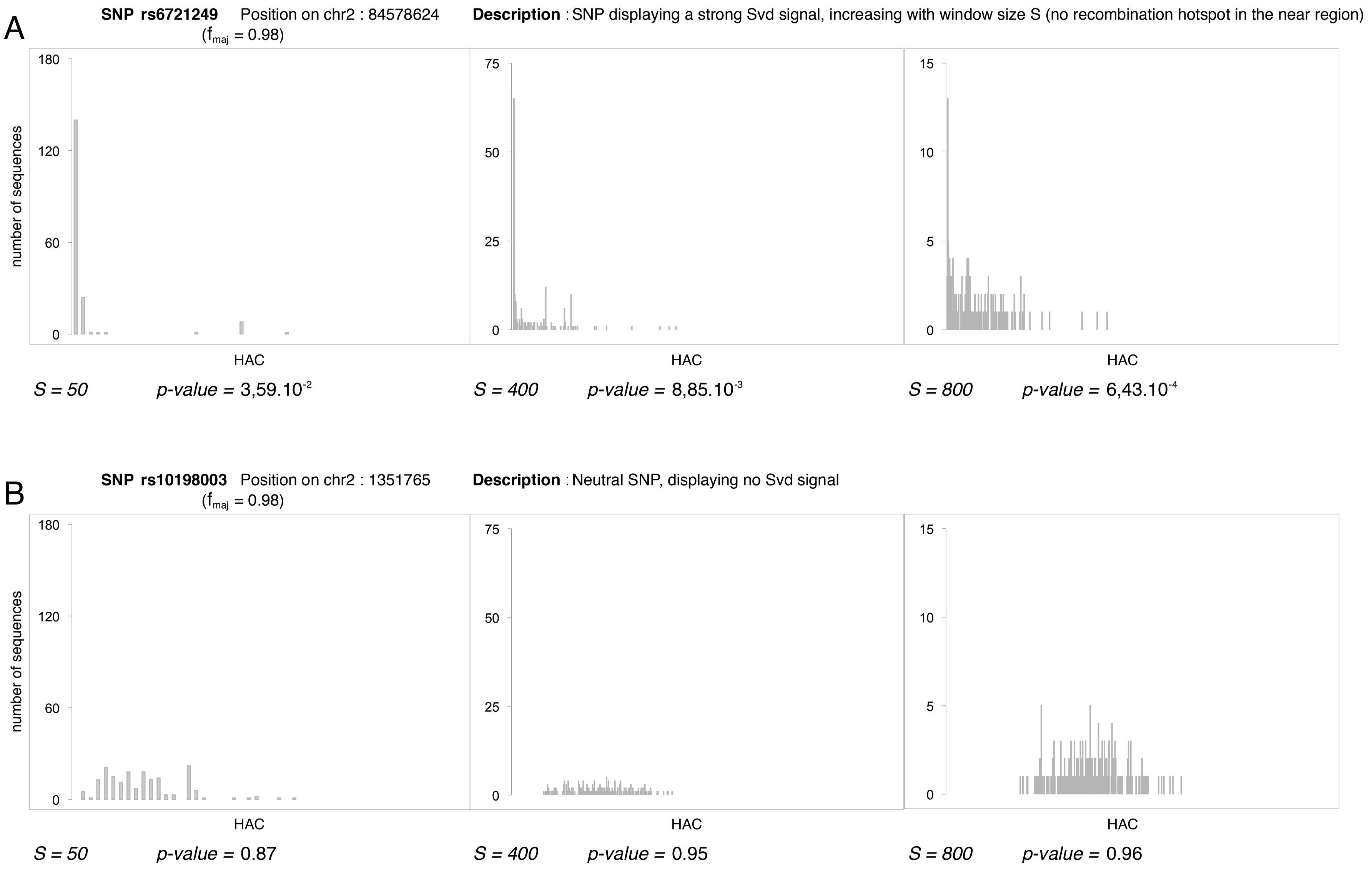

Figure S2 : Examples of Haplotype Allelic Classes distribution for sequences of size S carrying the major allele of the core SNP in HapMap ASI population.

C

SNP rs10166142 Position on chr2 : 135544054 (f_{maj} = 0.93) **Description** : SNP displaying Svd signal that appears with increasing window size S (no recombination hotspot in the near region)

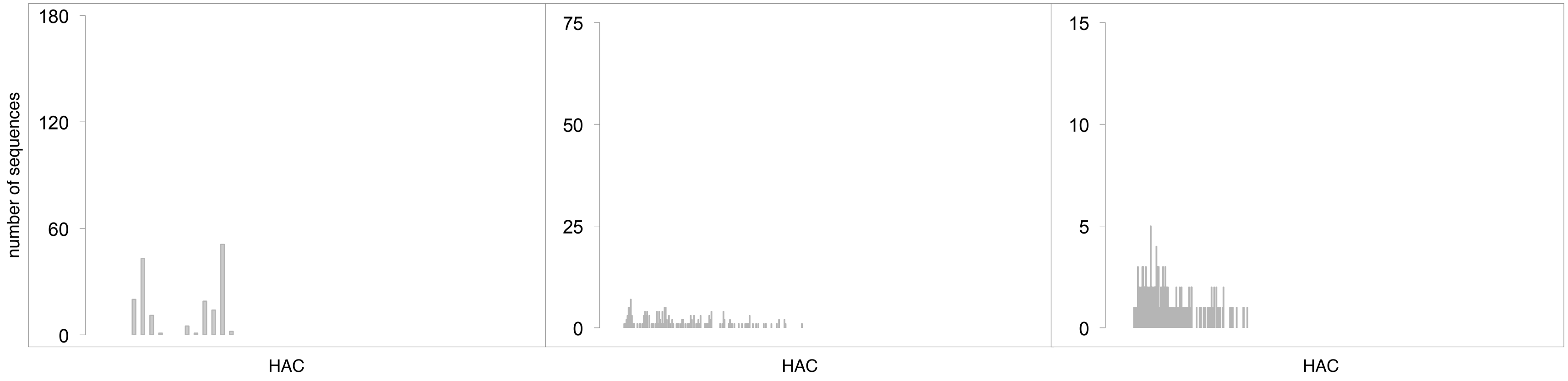

S = 50 *p-value* = 0,85 *S = 400* *p-value* = $1{,}37.10^{-2}$ *S = 800* *p-value* = $3{,}69.10^{-3}$

$7{,}68.10^{-2}$

D

SNP rs10803902 Position on chr2 : 177599185 (f_{maj} = 0.97) **Description** : SNP displaying Svd signal that fades with increasing window size S (presence of recombination hotspot in the near region)

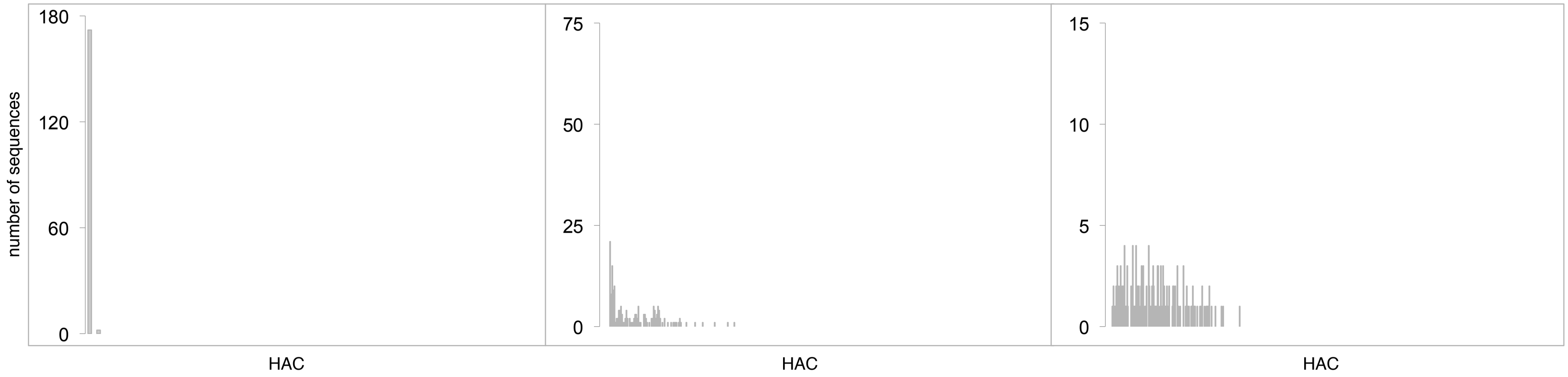

S = 50 *p-value* = $1{,}95.10^{-5}$ *S = 400* *p-value* = $2{,}08.10^{-4}$ *S = 800* *p-value* = $7{,}68.10^{-2}$

Figure S2 : Examples of Haplotype Allelic Classes distribution for sequences of size *S* carrying the major allele of the core SNP in HapMap ASI population.

Table S1 - List of the 26 SNPs from MCM6 locus from HapMap II CEU data.

Chr.2 position	Marker id	Alleles	Strand
136424478	rs3754689	A/**G**	-
136427890	rs2236783	**C**/T	-
136432949	rs3820790	**A**/T	-
136437008	rs3754686	**A**/G	-
136437098	rs3769005	**C**/G	-
136441435	rs4988243	**A**/G	-
136441963	rs4954490	A/**G**	+
136442378	rs4988235	**C**/T	-
136443052	rs2304369	A/**G**	+
136443403	rs4988232	**C**/T	-
136444330	rs4988226	C/**T**	-
136447193	rs4988218	**A**/G	-
136447987	rs309180	A/**G**	+
136448954	rs3213871	**C**/T	+
136452239	rs4988201	C/**T**	-
136453476	rs4988191	G/**T**	-
136454689	rs4988189	**A**/G	-
136455590	rs4988186	A/**C**	-
136455673	rs4988185	A/**G**	-
136455948	rs309176	C/**T**	+
136456679	rs4988178	C/**T**	-
136458046	rs3087348	**G**/T	-
136458114	rs4988173	A/**G**	-
136458418	rs4988172	C/**G**	-
136458679	rs1435577	**C**/G	+
136459810	rs3769001	C/**T**	-

The purported ancestral allele is presented in blue.

Table S2 - Detection power of Svd and iHS when the selected site is surrounded by recombination hotspots

Model	Svd		|iHS|	
	Critical value	Power	Critical value	Power
2 weak hotspots	2.10	0.72	3.90	0.27
2 strong hotspots	1.97	0.38	3.90	0.19
1 strong hotspot	2.13	0.67	4.01	0.17

Power to detect an ongoing sweep is reported for the two statistics on simulated data. The statistics are computed on n = 50 sequences of 100 Kb simulated under the selection scenarios with Θ = 223, with 1 or 2 hotspots, weak (10 times the recombination rate background ρ = Θ/2) or strong (100 times the recombination rate background ρ = Θ/2). The selected site is at a frequency of f = 0.9. In the 1-hotspot model, the selected site is situated at position 33 000 and the hotspot is situated between 66 and 67Kb. In the 2-hotspots model, the selected site is situated at position 50 000 and the hotspots are situated between 32 and 33Kb and 66 and 67Kb. Power is measured at $p = 0.05$, critical values are obtained using identical simulations with $s = 0$. The normalized value of Svd and the absolute value of the unstandardized iHS statistic are used.
